# Optimum maternal healthcare service utilization and infant mortality in Ethiopia

**DOI:** 10.1186/s12884-021-03860-z

**Published:** 2021-05-19

**Authors:** Girmay Tsegay Kiross, Catherine Chojenta, Daniel Barker, Deborah Loxton

**Affiliations:** 1 Department of Public Health, College of Health Sciences, Debre Markos University, Debre Markos, Ethiopia; 2 Research Centre for Generational Health and Ageing, Faculty of Health and Medicine, University of Newcastle, Newcastle, New South Wales Australia; 3 School of Medicine and Public Health, Faculty of Health and Medicine, University of Newcastle, Newcastle, New South Wales Australia

**Keywords:** Maternal healthcare utilization, Infant mortality, Ethiopia

## Abstract

**Background:**

Ethiopia has one of the highest rates of infant mortality in the world. Utilization of maternal healthcare during pregnancy, at delivery, and after delivery is critical to reducing the risk of infant mortality. Studies in Ethiopia have shown how infant survival is affected by utilization of maternal healthcare services, however, no studies to date have investigated the relationship between optimum utilization of maternal healthcare services utilization and infant mortality. Therefore, this study examined the effect of optimum utilization of maternal healthcare service on infant mortality in Ethiopia based on the World Health Organization (WHO, 2010) guidelines.

**Methods:**

We used nationally representative cross-sectional data from the Ethiopian Demographic and Health Survey (EDHS). Sampling weights were applied to adjust for the non-proportional allocation of the sample to the nine regions and two city administrations as well as the sample difference across urban and rural areas. A total of 7193 most recent births from mothers who had provided complete information on infant mortality, ANC visits, tetanus injections, place of delivery and skilled birth attendance during pregnancy were included. The EDHS was conducted from January to June 2016. We applied a multivariate logistic regression analysis to estimate the relationship between optimum maternal healthcare service utilization and infant mortality in Ethiopia.

**Results:**

The findings from this study showed that optimum maternal healthcare service utilization had a significant association with infant mortality after adjusting for other socioeconomic characteristics. This implies that increased maternal healthcare service utilization decreases the rate of infant mortality in Ethiopia. The main finding from this study indicated that infant mortality was reduced by approximately 66% among mothers who had high utilization of maternal healthcare services compared to mothers who had not utilized maternal healthcare services (AOR = 0.34; 95%CI: 0.16–0.75; *p*-value = 0.007). Furthermore, infant mortality was reduced by approximately 46% among mothers who had low utilization of maternal healthcare services compared to mothers who had not utilized any maternal healthcare services (AOR = 0.54; 95%CI: 0.31–0.97; *p*-value = 0.040).

**Conclusions:**

From this study, we concluded that optimum utilization of maternal healthcare services during pregnancy, at delivery and after delivery might reduce the rate of infant mortality in Ethiopia.

## Background

Infant mortality is defined as the number of deaths of children under 1 year of age per 1000 live births in the same year [1]. It is an essential national health indicator; a high rate of infant mortality may be an indicator of poor healthcare infrastructure or poor health services [1, 2]. A reduction in infant mortality is one of the priorities of global health and international development agendas [3, 4]. It was a priority area during the MDGs, and it remains as a priority area in the SDGs for 2030 [5, 6]. Over the past two decades, the world has achieved remarkable progress in child survival [4]. Globally, the IMR has decreased by 50% between 1990 and 2017 [4]. According to a 2017 WHO report, infant mortality accounts for 75% of all under-5 deaths [4]. The risk of a child dying before completing their first year of life was six times higher in the African region than that in the European region [4]. Infant deaths in developing countries are mainly due to preventable causes, such as pneumonia and diarrhoea [7]. The majority of infant deaths are due to avoidable causes and can be prevented using basic maternal healthcare service interventions, including the utilisation of prenatal care services, skilled delivery and postnatal care [8–10]. Despite the government’s considerable efforts to decrease the number of infant deaths, the rate of infant mortality in Ethiopia remains among the highest in the world [11, 12]. One of the key reasons for high infant mortality in Ethiopia is the low utilisation of modern healthcare services by many women in Ethiopia [13]. Previous cross-sectional studies based on DHS data in Ethiopia have clearly indicated that utilisation of maternal health services is very low in the country [14].

According to results of the Global Burden of Disease Study in 2015, achieving the SDG targets regarding maternal and child mortality by 2030 will require 91% coverage of one ANC visit, 78% of four ANC visits, 81% of in-facility delivery and 87% of skilled birth attendance [15]. In Ethiopia ANC utilisation coverage, skilled delivery and postnatal care utilisation were 35, 62, 28 and 17%, respectively [16]. Even though home delivery remains high, primarily in hard-to-reach rural areas, there have been improvements in ANC service utilisation and institutional delivery in Ethiopia [16].

A large global health study reported that half of the world’s population do not have access to modern health services [17]. However, access is one of the basic principles of universal health service coverage [18]. The United Nations General Assembly has called on all countries to ensure universal health service coverage by 2030 [19]. Ethiopia is one of the countries with a high disease burden of reproductive health problems, maternal health problems, neonatal and child health problems and infectious diseases problems [20]. Additionally, the country has low basic health service coverage and low health service utilisation [21]. In 2015, the national health service coverage was 34.3%, ranging from 52.2% in the Addis Ababa city administration to 10% in the Afar region; the universal health service coverage for neonatal and child health was 37.5% [22].

In this study, optimum maternal healthcare service utilisation is defined as the utilisation of WHO-recommended ANC visits, tetanus injection, skilled delivery, place of delivery and postnatal checks. The WHO recommends that a pregnant woman should receive a minimum of four ANC visits [23]. According to the WHO, the essential components of a basic prenatal care program are the identification of pregnancy, management of pregnancy-related complications, treatment of underlying illnesses, disease screening, preventive measures including tetanus toxoid immunisation, de-worming, iron and folic acid, treatment of malaria in pregnancy, a birth and emergency preparedness plan, promotion of healthy behaviours at home, helping the pregnant woman and her partner prepare emotionally and physically for birth and care of their baby, and the promotion of postnatal family planning/birth spacing [23].

Poor utilisation of maternal healthcare services has a significant impact on child health outcomes [24]. Utilisation of recommended ANC during pregnancy improves maternal and child health outcomes [25]. Evidence from DHS data from 17 SSA countries showed that having prenatal care provided by a skilled practitioner reduced the odds of neonatal mortality by 30% [26]. Findings from a systematic review in Ethiopia also showed that ANC utilisation reduced neonatal mortality by 34% [27, 28]. Having optimum ANC follow-up for either chronic or acute diseases during pregnancy can prevent further complications occurring for mothers and infants and reduce the prevalence of low birth weight by improving the nutritional status of a mother during pregnancy [29–31].

Tetanus immunisation during pregnancy has been associated with a reduction in infant mortality; providing women with two doses of tetanus toxoid was estimated to reduce mortality from neonatal tetanus by 94% [32]. The deaths of early infants caused by neonatal tetanus can also be decreased by half through a combination of maternal tetanus immunisation and clean delivery practices [33].

Previous studies have found an association between the deaths of children less than 1 year old and skilled birth and health facility delivery [34]. A systematic review from developing countries showed that delivery at health facilities reduced the risk of under-1 child mortality by 29% [34]. A systematic review conducted in developing countries found that frequent postnatal visits reduced neonatal deaths; it was also encouraged that women breastfeed exclusively [35].

Infant mortality is significantly associated with the number of ANC visits, tetanus injections during pregnancy, the place of delivery and skilled birth attendance, among other variables. Maternal healthcare service utilisation during pregnancy and delivery decreases the risk of infant mortality. However, independently examining the relationships between infant mortality and each of ANC visits, postnatal care and tetanus vaccination might not give adequate evidence for policy formulation. For example, if a pregnant woman has attended the recommended ANC, but was not assisted by a skilled health professional and did not follow up on postnatal care, the survival of her child may be at higher risk.

The interrelated nature of these maternal healthcare service indicators needs to be addressed in an integrated way to enhance the survival chances of infants. Therefore, in this study, five indices of maternal healthcare services were combined by scoring each indicator to produce a combined score. Combining the indices of maternal healthcare service utilisation can help in understanding the effects of optimum maternal healthcare service utilisation on infant mortality in Ethiopia. We expected that the rate of infant mortality would be lower among women who had a high utilisation of maternal healthcare services, compared to women who had no experience of maternal healthcare service utilisation.

## Methods

### Study area and setting

We used data from the fourth EDHS, which was conducted from January 18, 2016, to June 27, 2016. The EDHS was conducted by the Central Statistics Agency in collaboration with the Federal Ministry of Health and the Ethiopian Public Health Institute with technical assistance from ICF International and funding from the United States Agency for International Development. The study was conducted in all nine geographical regions and two administrative cities of Ethiopia [16].

### Study design and sampling

The 2016 EDHS is a nationally representative cross-sectional household survey. Study participants were selected through a stratified two-stage cluster sampling technique. The sampling frame used for the 2016 EDHS was the Ethiopia Population and Housing Census, which was conducted in 2007 by the Ethiopian Central Statistics Agency. It contained information about the EA location, type of residence (urban or rural) and estimated number of residential households. The 2016 EDHS sample was stratified and selected in two stages [16]. In the first stage, a total of 645 EAs (202 in urban areas and 443 in rural areas) were selected with probabilities proportional to EA size (based on the 2007 Population and Housing Census). In the second stage, 28 households were selected per cluster with equal-probability systematic sampling. All women aged 15–49 years who were either permanent residents of the selected households or visitors who stayed in the household the night before the survey were eligible to be interviewed [16]. In the original sample, 15,683 women of child-bearing age who had live births within the 5 years prior to the survey were interviewed [16]. Out of 15,683 women of child-bearing 10,641 women who had live births within the 5 years were selected. Finally, a total of 7193 most recent births who had provided complete information on infant mortality, ANC visits, tetanus injections, place of delivery and skilled birth attendance during pregnancy were included.

### Study variables

The outcome variable for this study was infant death (either ‘yes’ or ‘no’).

### Data analysis

Data were analysed using Stata version 15. The data were weighted before use to ensure representativeness, since a cluster design approach was used. Data were cleaned for completeness, and descriptive statistics are presented.

At the first stage of the analysis, a chi-square model was used to establish an association between infant mortality and the independent variables mentioned in Table 1. Additionally, a chi-square model was used to establish an association between maternal healthcare service utilisation and other independent variables to identify confounding variables. Thereafter, statistically significant variables (α < 5%) associated with infant mortality and associated with maternal healthcare service utilisation were entered into a multivariate model to account for confounding. Finally, multivariate logistic regression analysis was performed to estimate the AORs. The association between maternal healthcare service utilisation and infant mortality was expressed using AORs with 95% confidence intervals. A *p* value of .05 was used as the cut-off for statistical significance.

**Table 1:** Description and Measurement of Exposure Variables

**Explanatory variables**	
Optimum maternal healthcare service utilisation	The principal independent variable was ‘maternal healthcare service utilisation’. It was generated using the variables ‘antenatal care visit’, ‘tetanus vaccination’, ‘place of delivery’, ‘skilled birth attendance’ and ‘postnatal check-up’. Scores were assigned to the responses of each woman to each of the questions: number of antenatal care visits (none = 0; 1–3 = 1; ≥4 = 2), skilled delivery (no = 0; yes = 1), postnatal check (no = 0; yes = 1), received the required number of tetanus injections during pregnancy (no = 0; yes = 1), place of delivery (home = 0; other facilities = 1; modern health facility = 2). This produced a maximum overall score of 7 and a minimum of 0. Following this, the overall total score for eligible respondents was disaggregated by quartile. 2016 Ethiopian Demographic and Health Survey Data were collected health-related information five years before the survey that means all the data were collected before WHO introduced the new recommended antenatal visits. $$ \kern1.25em MCHI=\kern2.5em \left\{\begin{array}{c} None, if\kern27.75em x=0\kern1.25em \\ {} Low, if\kern8.5em 0<x<50\% of\ the\ \mathit{\max}. overall\ score\kern5em \\ {} Medium, if\kern9.25em 50\%\le x<75\% of\ the\ \mathit{\max}. overall\ score\\ {} High, if\kern10em 75\%\le x<100\% of\ the\ \mathit{\max}. overall\ score\ \end{array}\kern1em \right. $$
Infant sex	Categorised as ‘male’ or ‘female’.
Preceding birth interval	This is the difference in months between the current birth and the previous birth, counting twins as one birth; it was based on the date of birth of the children based on the mother’s self-reporting [36, 37]. It was re-coded as (i) ‘less than 24 months (or short birth interval)’, (ii) ‘between 24 and 59 months (or recommended birth interval)’ and (iii) ‘greater than 60 months’ [36, 37].
Reported birth size	Mothers were asked to estimate the size of their child based on their experience or by comparison with a previous child. The response options were ‘greater than average’, ‘average’, ‘smaller than average’ and ‘very small’. This variable was re-coded as ‘above average’, ‘average’ and ‘below average’ (smaller than average or very small) [36, 37].
Multiple births	This is the number of births for one pregnancy, and the response option was open to any number of births. This was re-coded as ‘single birth’ for one birth or ‘multiple birth’ for more than one birth [36, 37].
Total live births	Participants were asked how many live births they had had in their lives, and the response option was continuous. This was re-coded based on quartiles as (i) ‘one live birth’, (ii) ‘two to three live births’, (iii) ‘four to five live births’ and (iv) ‘more than five live births’. If the respondent was pregnant during the interview, one was added to the total live births [36, 37].
Maternal age	Women were asked their age (in years) when completing the survey. It was then categorised as ‘15–19’, ‘20–24’, ‘25–29’, ‘30–34’, ‘35–39’, ‘40–44’ and ‘45+’. F For this study, the reproductive age of the mother was re-coded as ‘young’ (15–24 years), ‘young adult’ (25–34 years) and ‘middle aged’ (35–49 years) to obtain adequate samples for each category [36, 37].
Religion	Participants were asked to report their religion as either ‘Orthodox’, ‘Catholic’, ‘Protestant’, ‘Muslim’ or ‘Traditional or other’. Orthodox, Catholic and Protestant were re-categorised as ‘Christian’; the categories of ‘Muslim’ and ‘Traditional or other’ were retained [36, 37].
Maternal education	The original question was ‘what is your highest level of education?’, and the response options were ‘no education’, ‘primary education’, ‘secondary education’ and ‘higher education’. We re-coded these as ‘no education’, ‘primary education’ and ‘secondary education and above’. We merged secondary and higher education because of the small numbers in the higher education category [36, 37].
Decision-making autonomy	The original questions were ‘who usually decides to obtain healthcare?’, ‘who usually decides on the purchase of household items?’ and ‘who usually decides to visit relatives?’. The response options were ‘respondent’ or ‘partner’. A new variable was created as a combination of the three responses. The value of each characteristic was either 0 (partner) or 1 (respondent). If the respondent scored 3 out of 3, they were categorised as ‘yes’ to having decision-making autonomy and ‘no’ if otherwise [36, 37].
Media exposure	The original questions for this variable were ‘do you listen to the radio at least once a week, less than once a week or not at all?’, ‘do you read a newspaper/magazine at least once a week, less than once a week or not at all?’ and ‘do you watch television at least once a week, less than once a week or not at all?’ [37]. A composite variable was created combining whether a respondent read newspapers/magazines, listened to the radio and/or watched TV. This was categorised as ‘no access’ if the woman had no access to any of the three media, ‘1’ if the woman had access to any of the three media less than once a week, and ‘2’ if the woman had access to any of the three media at least once a week [36, 37].
Number of pregnancy losses (miscarriage, abortion or stillbirth) and child deaths	Participants were asked if they ever had a pregnancy that miscarried, was aborted or ended in a stillbirth, and if they had ever successfully given birth to a child who later died. A new variable was created as a combination of child mortality (from the birth history question) and pregnancy loss (from the pregnancy history question). To generate this new variable, the number of child deaths from the birth history was first generated. Second, the number of pregnancy losses (miscarriage, abortion or stillbirth) was generated. The status of the index child was excluded from the calculation. Finally, the above two values were added to create the new variable with the following categories: ‘no adverse pregnancy events’, ‘one adverse pregnancy event’, ‘two adverse pregnancy events’ and ‘three or more adverse pregnancy events’ [36, 37].
Sex of the household head	Categorised as ‘male’ or ‘female’.
Household wealth index	In the EDHS, wealth index was calculated based on household assets, such as televisions and bicycles. Principal components analysis was applied to generate the wealth index as a continuous scale of relative wealth. Wealth index was categorised into five wealth quintiles: ‘very poor’, ‘poor’, ‘middle’, ‘rich’ and ‘very rich’. For this analysis, we re-coded the wealth index as three categories for adequate sampling in each category: as ‘poor’ (poor and very poor), ‘middle’ and ‘rich’ (rich and very rich) [36, 37].
Distance to health facilities	Distance to health facilities was based on participants’ subjective ratings. The original response options were ‘distance to health facilities is a big problem’ or ‘distance to health facilities is not a big problem’ [36, 37].
Place of residence	This was originally recorded as ‘rural’ or ‘urban’ and not changed for this analysis [36, 37].
Region of residence	Defined as the region in which the infant’s mother was raised. The variable was re-coded as ‘agrarian’ (encompassing Tigray, Amhara, Oromia, Benishangul, SNNPR, Gambela and Harari), ‘pastoralist’ (Afar and Somali) or ‘city dweller’ (Addis Ababa and Dire Dawa). An agrarian society is any community whose economy is based on producing and maintaining crops and farmland. A pastoralist society is any community whose economy is based on raising livestock. A city-dweller society is any city community [36, 37].
Maternal employment	Women were considered to be employed if they had done any work other than housework in the 12 months prior to the survey and if they were paid for their labour in cash or in kind. The response options were ‘employed for cash’, ‘employed not for cash’ and ‘unemployed’. This was re-categorised as ‘employed’ and ‘not employed’.

MCHI, *EDHS* Ethiopian Demographic and Health Survey, *SNNPR* Southern Nations, Nationalities and Peoples’ Region

## Results

The rate of infant mortality was higher among boys than girls: 23.8 per 1000 and 8.6 per 1000, respectively. Of all infant deaths, 73.6% were male. The rate of infant mortality in rural areas was 29.5 per 1000. The rate of infant mortality was higher in rural areas than in urban areas (2.9 per 1000). The rate of infant mortality decreased when maternal education increased. The rate of infant mortality was higher among infants born with a small birth size compared to infants born with an average birth size. The rate of infant mortality was higher across women who had not used maternal healthcare services, compared with women who had high maternal healthcare service use (see Tables 2 and 3).

**Table 2:** Frequency and percentage distribution of infant mortality by study population characteristics

Variable	Infant mortality		Total births	IMR (per 1000)
	Yes (***n*** = 246)	No (***n*** = 7344)		
Infant sex				
Male	181 (73.6)	3759 (51.2)	3940 (51.9)	23.8
Female	65 (24.4)	3585 (48.8)	3650 (48.1)	8.6
Type of birth				
Singleton	213 (86.6)	7257 (98.8)	7470 (98.4)	28.1
Multiple	33 (13.4)	87 (1.2)	120 (1.6)	4.3
Reported birth weight				
Above average	86 (35.0)	2313 (31.5)	2399 (31.6)	11.3
Average	78 (31.7)	3069 (41.8)	3147 (41.5)	10.3
Below average	82 (33.3)	1962 (26.7)	2044 (26.9)	10.8
Preceding birth interval				
First birth	38 (15.4)	1408 (19.2)	1446 (19.1)	5.0
< 24 months	56 (22.8)	1020 (13.9)	1076 (14.2)	7.4
24–59 months	118 (48.0)	3857 (52.5)	3975 (52.4)	15.5
≥ 60 months	34 (13.8)	1057 (14.4)	1091 (14.4)	4.5
Maternal age (years)				
15–24	58 (23.6)	1747 (23.8)	1805 (23.8)	7.6
25–34	110 (44.7)	3716 (50.6)	3826 (50.4)	14.5
35–49	78 (31.7)	1881 (25.6)	1959 (25.8)	10.3
Maternal education				
No education	161 (65.4)	4630 (63.0)	4791 (63.0)	21.2
Primary education	67 (27.2)	2082 (28.3)	21,491 (28.3)	8.8
Secondary education and above	18 (7.3)	632 (8.6)	650 (8.7)	2.4
Age at first birth (years)				
Less than 20	23 (9.3)	666 (9.1)	689 (9.1)	3.0
20–34	160 (65.1)	5278 (71.9)	5438 (71.6)	21.1
35–49	63 (25.6)	1400 (19.1)	1463 (19.3)	8.3
Total children ever born				
1	30 (12.2)	1405 (19.1)	1435 (18.9)	4.0
2–3	83 (33.7)	2198 (29.9)	2281 (30.0)	10.9
4–5	36 (14.6)	1715 23.4)	1751 (23.1)	4.7
5+	97 (39.4)	2025 (27.6)	2122 (38.0)	12.8
Wealth index				
Poor	107 (43.5)	3199 (43.6)	3306 (43.6)	14.1
Middle	46 (18.7)	1542 (21.0)	1588 (20.9)	6.1
Rich	93 (37.8)	2603 (35.4)	2696 (35.5)	12.3
Religion				
Christian	117 (47.6)	4489 (61.1)	4605 (60.1)	15.4
Muslim	129 (52.4)	2695 (36.7)	2824 (37.2)	17
Traditional	0 (0.0)	97 (1.3)	97 (1.3)	0
Other	0 (0.0)	64 (0.9)	64 (0.8)	0
Number of living children				
One or less	82 (33.3)	1503 (20.5)	1585 (20.9)	10.8
2–3	62 (25.2)	2405 (32.7)	2467 (32.5)	8.2
4–5	46 (18.7)	1837 (25.0)	1883 (24.8)	6.1
≥ 6	56 (22.8)	1599 (21.8)	1655 (21.8)	7.4
Number of pregnancy losses (miscarriage, abortion or stillbirth) and child deaths				
None	83 (33.7)	5229 (71.2)	5312 (70.0)	10.9
1	93 (37.8)	1435 (19.5)	1528 (20.1)	12.3
2	49 (19.9)	452 (6.2)	501 (6.6)	6.5
≥ 3	21 (8.5)	228 (3.1)	249 (3.3)	2.8
Head of household				
Male	215 (87.4)	6259 (85.2)	6474 (85.3)	28.3
Female	31 (12.6)	1085 (14.8)	1116 (14.7)	4.1
Region				
Tigray	12 (4.9)	525 (7.1)	537 (7.1)	1.6
Afar	3 (1.2)	68 (0.9)	71 (1.0)	0.4
Amhara	49 (19.9)	1584 (21.6)	1633 (21.5)	6.5
Oromia	118 (48.0)	3, 011 (41.0)	3129 (41.2)	15.5
Somali	13 (5.3)	256 (3.5)	269 (3.5)	1.7
Benishangul	2 (0.8)	79 (1.1)	81 (1.1)	0.3
SNNPR	43(17.5)	1557 (21.2)	1600 (21.1)	5.7
Gambela	1 (0.4)	20 (0.3)	21 (0.3)	0.1
Harari	1 (0.4)	17 (0.2)	18 (0.2)	0.1
Addis Ababa	3 (1.2)	195 (2.7)	198 (2.6)	0.4
Dire Dawa	1 (0.4)	32 (0.4)	33 (0.4)	0.1
Residence				
Urban	22 (8.9)	947 (12.9)	969 (12.8)	2.9
Rural	224 (91.1)	6397 (87.1)	6621 (87.2)	29.5
Maternal decision-making autonomy				
No	112 (45.5)	2563 (34.9)	2675 (35.2)	14.8
Yes	134 (54.5)	4781 (65.1)	4915 (64.8)	17.7
Maternal employment				
Employed	122 (49.6)	3957 (53.9)	4079 (53.7)	16.1
Unemployed	124 (50.4)	3388 (46.1)	3512 (46.3)	16.3
Access to media				
No access	138 (56.1)	4832 (65.8)	4970 (65.5)	18.2
Less than once a week	55 (22.4)	1080 (14.7)	1135 (15.0)	7.2
At least once a week	54 (22.0)	1432 (19.5)	1486 (19.5)	7.1
MHCI				
None	93 (38.8)	1931 (26.3)	2024 (26.7)	12.3
Low	89 (36.2)	3078 (41.9)	3167 (41.7)	11.7
Medium	36 (14.6)	996 (13.6)	1032 (13.6)	4.7
High	28 (11.4)	1339 (18.2)	1367 (18.0)	3.7
Contextual region				
Agrarian	225 (91.5)	6793 (92.5)	7018 (92.5)	29.6
Pastoralist	16 (6.5)	24 (4.4)	340 (4.5)	2.1
City dweller	5 (2.0)	227 (3.1)	232 (3.0)	0.7

*N* = 7590; values are given as *n* (%) except where noted*. IMR* infant mortality rate, *SNNPR* Southern Nations, Nationalities and Peoples’ Region, MHCI

**Table 3:** Comparison of the original sample and selected sample

Variable	Selected sample		Original sample	
	Infant mortality		Infant mortality	
	Yes (***n*** = 246)	No (***n*** = 7344)	Yes (***n*** = 495)	No (***n*** = 10,528)
Sex				
Male	181 (73.6)	3759 (51.2)	325 (65.7)	5400 (51.3)
Female	65 (24.4)	3585 (48.8)	170 (34.3)	5128 (48.7)
Types of births				
Singleton	213 (86.6)	7257 (98.8)	458 (92.5)	10,419 (99.0)
Multiple	33 (13.4)	87 (1.2)	37 (7.5)	109 (1.0)
Birth size				
Above average	86 (35.0)	2313 (31.5)	163 (33.0)	3323 (32.6)
Average	78 (31.7)	3069 (41.8)	172 (34.7)	4488 (42.6)
Below average	82 (33.3)	1962 (26.7)	160 (32.3)	2717 (25.8)
Birth interval				
First birth	38 (15.4)	1408 (19.2)	93 (18.8)	1977 (18.8)
< 24 months	56 (22.8)	1020 (13.9)	151 (30.5)	1891 (18.0)
24–59 months	118 (48.0)	3857 (52.5)	193 (39.0)	5552 (52.7)
≥ 60 months	34 (13.8)	1057 (14.4)	58 (11.7)	1208 (11.5)
Maternal age				
15–24	58 (23.6)	1747 (23.8)	114 (23.0)	2332 (22.2)
25–34	110 (44.7)	3716 (50.6)	249 (50.3)	5593 (53.1)
35–49	78 (31.7)	1881 (25.6)	132 (26.7)	2603 (24.7)
Maternal education				
No education	161 (65.4)	4630 (63.0)	341 (68.9)	6943 (65.9)
Primary education	67 (27.2)	2082 (28.3)	122 (24.6)	2829 (26.9)
Secondary education and above	18 (7.3)	632 (8.6)	32 (6.5)	756 (7.2)
Wealth index				
Poor	107 (43.5)	3199 (43.6)	218 (44.0)	4939 (46.9)
Middle	46 (18.7)	1542 (21.0)	98 (19.8)	2182 (20.7)
Rich	93 (37.8)	2603 (35.4)	179 (36.2)	3408 (32.4)
Residence				
Urban	22 (8.9)	947 (12.9)	51 (10.3)	1165 (11.1)
Rural	224 (91.1)	6397 (87.1)	444 (89.7)	9364 (88.9)
Access to media				
No access	138 (56.1)	4832 (65.8)	292 (59.0)	7084 (67.3)
Less than once a week	55 (22.4)	1080 (14.7)	102 (20.6)	1537 (14.6)
At least once a week	54 (22.0)	1432 (19.5)	101 (20.4)	1907 (18.1)
Contextual region				
Agrarian	225 (91.5)	6793 (92.5)	449 (90.7)	9660 (91.8)
Pastoralist	16 (6.5)	24 (4.4)	36 (7.3)	587 (5.6)
City dweller	5 (2.0)	227 (3.1)	10 (2.0)	281 (2.7)

According to the multivariate logistic regression model, infant mortality was significantly associated with multiple births, birth interval, number of pregnancy losses (miscarriage, abortion or stillbirth) or child deaths, and maternal healthcare utilisation. Women with multiple pregnancies had higher odds of infant mortality compared with women who had a single pregnancy, AOR = 11.7, 95% CI [5.45, 25.19], *p* < .001. Preceding birth interval was also significantly associated with infant mortality; an infant with a short birth interval had higher odds of death compared with an infant born with an interval of 24 to 59 months, AOR = 1.6, 95% CI [1.11, 2.s, *p* = .005. Other controlling variables associated with infant mortality included pregnancy loss and previous history of child deaths. As the number of pregnancy losses and previous child deaths increased, infant mortality also increased; infants born to women with more than three pregnancy-related events were 7.2 times more likely to die compared with infants born to mothers who had no pregnancy losses or child deaths, AOR = 7.16, 95% CI [2.38, 21.50, *p* < .001. Women’s access to modern healthcare services was significantly associated with deaths of children under 1 year; infants of women who had high access to modern healthcare services had lower odds of death compared with infants of women who had no access to modern healthcare services, AOR = 0.34, 95%CI [0.16, 0.75], *p* = .007 (see Table 4).

**Table 4:** Multivariate logistic regression association of maternal healthcare service utilisation with infant mortality in Ethiopia

Variable	COR (95% CI)	AOR (95% CI)	***p***
Contextual region			
Agrarian	1 (reference)	1 (reference)	
Pastoralist	1.57 (1.17, 2.12)	1.23 (0.77, 1.96)	0.39
City dweller	0.86 (0.53, 1.40)	0.95 (0.39, 2.33)	0.91
Multiple births			
Yes	9.8 (6.17, 15.71)	11.71 (5.45, 25.19)*	< 0 .001
No	1 (reference)	1 (reference)	
Birth interval			
First birth	1.40 (1.01, 1.98)	1.40 (0.67, 2.94)	0.37
< 24 months	1.62 (1.15, 2.31)	1.60 (1.11, 2.60)*	0.01
24–59 months	1 (reference)	1 (reference)	
≥ 60 months	1.27 (0.84, 1.92)	1.30 (0.68, 2.44)	0.43
Maternal education			
No education	1 (reference)	1 (reference)	
Primary education	0.97 (0.67, 1.23)	1.21 (0.72, 2.04)	0.46
Secondary or higher	0.65 (0.41, 1.05)	1.54 (0.46, 5.20)	0.49
Maternal age (years)			
15–24	1 (reference)	1 (reference)	
25–34	0.7 (0.51, 0.97)	0.64 (0.35, 1.16)	0.14
35–49	1.01 (0.72, 1.44)	0.54 (0.25, 1.16)	0.12
Wealth index			
Poor	1 (reference)	1 (reference)	
Middle	0.65 (0.43, 1.01)	0.92 (0.47, 1.82)	0.82
Rich	0.66 (0.48, 0.89)	1.39 (0.83, 2.31)	0.21
Residence			
Urban	1 (reference)	1 (reference)	
Rural	1.78 (1.21, 2,63)	1.20 (0.49, 2.93)	0.69
Maternal decision-making autonomy			
No	1 (reference)	1 (reference)	
Yes	0.79 (0.61, 1.64)	0.70 (0.45, 1.07)	0.10
Number of pregnancy losses (miscarriage, abortion or stillbirth) and child deaths			
None	1 (reference)	1 (reference)	
1	4.05 (2.98, 5.49)	4.65 (2.86, 7.60)*	< 0.001
2	5.59 (3.75, 8.33)	7.82 (4.18, 14.66)*	< 0 .001
≥ 3	4.85 (2.90, 8.12)	7.16 (2.38, 21.50)*	< 0 .001
MHCI			
None	1 (reference)	1 (reference)	
Low	0.59 (0.44, 0.82)	0.54 (0.31, 0.97)*	0.04
Medium	0.57 (0.37, 0.88)	0.78 (0.41, 1.50)	0.46
High	0.44 (0.30, 0.65)	0.34 (0.16, 0.75)*	0.01

*AOR* adjusted odds ratio, MHCI

* *p* < .05. ** *p* < .001

## Discussion

The rate of infant mortality was lower among women who experienced high utilisation of maternal healthcare services compared with women who had no experience of maternal healthcare service utilisation: 3.7 and 12.3 per 1000, respectively. These rates of infant deaths are consistent with a previous study conducted in Nigeria, which reported that the mean maternal healthcare service utilisation was higher among mothers who reported low infant deaths compared to mothers who reported high infant deaths [38].

The results from the multivariate analysis in this study showed that the combined five indicators of maternal healthcare service utilisation (antenatal attendance, tetanus injection, place of delivery, skilled birth attendance and postnatal check) were significantly associated with infant deaths. The findings from this study showed that optimum maternal healthcare service utilisation had a significant association with infant mortality: as maternal healthcare service utilisation increased, infant mortality decreased. Having high maternal healthcare service utilisation prevented infant mortality by approximately 66% compared to having no maternal healthcare service utilisation. Additionally, having low maternal healthcare service utilisation prevented infant mortality by 46% compared to having no maternal healthcare service utilisation at all. Previous studies have reported that high maternal healthcare service utilisation during pregnancy is essential for mothers’ health and the survival of their infants [26, 39]. Optimum maternal healthcare service utilisation during and after pregnancy is critical for the survival of an infant; early and regular maternal healthcare service utilisation is also essential for the health of both the mother and the infant [40]. Optimum maternal healthcare service utilisation, such as having the recommended number of ANC visits and receiving appropriate and timely care, ensures the survival of child [41]. While many previous studies have shown how infant mortality is affected by independent maternal healthcare service utilisation, examining independent relationships might not give adequate evidence regarding how maternal healthcare utilisation affects infant mortality [42, 43]. An infant of a woman who attended ANC but was not assisted by a skilled health professional during delivery may have reduced odds of survival. The evidence on combined indices of maternal healthcare service utilisation may provide clearer insight to policymakers in Ethiopia. The findings from this study may also contribute to the current literature on how combined maternal healthcare services could affect infant mortality.

Access to and utilisation of healthcare facilities during pregnancy can increase the likelihood of a woman using skilled birth attendance during delivery and postnatal check-up, which are essential to the survival of the infant. This finding is consistent with the literature on the association between maternal healthcare service utilisation and infant survival. The rate of low maternal healthcare service utilisation in Ethiopia is one of the highest in the world, which is also one of the contributing factors to high infant mortality in the country [44]. In a birth-cohort and matched case–control study in Ethiopia, it was reported that utilising ANC reduced infant mortality [45, 46]. This could be because mothers who attended ANC during pregnancy could properly receive services such as nutritional counselling, nutritional screening and more [47]. Maternal healthcare service utilisation may help to inform and educate mothers about maternal and infant health. It can provide an opportunity to screen for warning signs of pregnancy complications and to treat infections [27]. Additionally, maternal healthcare service use enables health workers to teach women about complications during pregnancy, labour and delivery [48]. Furthermore, ANC utilisation provides an opportunity to inform women about danger signs and the importance of modern healthcare utilisation [48].

A previous study investigating the combined effects of maternal healthcare service utilisation on infant mortality in Nigeria showed that infant deaths were higher among women with no or low maternal healthcare service utilisation compared to those with high and complete maternal healthcare service utilisation [38]. A national study in Kenya showed that a lack of skilled ANC and insufficient ANC visits increased neonatal mortality. In the same study, researchers also indicated that a single tetanus toxoid vaccination during pregnancy could prevent about 10% of neonatal deaths [49].

Prenatal healthcare service utilisation during pregnancy is critical for the fetus’s and the mother’s health. It also increases the chance of using a skilled birth attendant, which improves the likelihood of child survival in the first year of life [42]. ANC provides an opportunity to identify risk factors, prevent complications and improve the birth preparedness of pregnant women to reduce infant mortality [50]. In a national study in Zimbabwe, it was indicated that mothers’ health-seeking behaviours, such as giving birth in health facilities, receiving ANC from skilled providers and being assisted by skilled birth attendants during delivery, lowered the risk of child deaths at an early age. The results indicated that a one-unit increase in the quality of prenatal care lowered infant mortality by approximately 31% [51]. Studies conducted in poor resource countries have indicated that the utilisation of maternal healthcare services, such as skilled birth attendance and delivery at healthcare facilities, can reduce maternal and child mortality [52, 53]. A high coverage of healthcare service utilisation could reduce the death of a child: an evidence-based cost-effective intervention showed that a 90% coverage of facility-based care could reduce early child mortality by 23–50% [43]. Another study in West Africa showed that having any prenatal care was associated with lower infant mortality [54].

Maternal healthcare service utilisation may influence infant outcomes in different ways. For example, prenatal care may make a difference in pregnancy outcomes [39, 55]. Inadequate or lack of prenatal care is a risk factor for low birth weight and other poor pregnancy outcomes. Women who have no access to maternal healthcare services are more likely to give birth to a low-weight infant [39, 55]. Access to good health care during pregnancy is essential for a woman’s health and for the development of her unborn child. Additionally, accessing a modern health system during pregnancy increases the chance of using a skilled birth attendant during delivery, and this may contribute to the good health of a child in their first year of life [40, 42]. Women from low-income countries, such as those in SSA and South Asia, are less likely to receive adequate maternal health care; these low-income regions have low numbers of skilled health workers [56]. By contrast, in high-income and middle-income countries, more than 90% of all births are assisted by a trained midwife, doctor or nurse; in several low-income countries, less than half of all births are assisted by such skilled health personnel [56]. In addition to the maternal healthcare utilization, multiple birth babies, history of pregnancy loss and child death was significantly associated with infant mortality. For example, in a study of a health survey of 7001 live births in Bangladesh, infants born to mothers who had multiple birth babies were about six times more likely to die than those born singleton [139]. The possible reason for high risk of death among the multiple births may be due to the economic burden of the family, and this affects the quality of nutrition and health care of children [74, 143].

This study has the following strengths and limitations. The use of a nationally representative dataset that covered all regions in Ethiopia may help to provide nationwide evidence to policymakers. The EDHS data were also well designed. There are, however, also some limitations, such as recall bias, because study participants were asked to remember events up to 5 years before the survey, and most of the data were collected based on self-reports, which may be subject to error. There may be also potential selection bias due to missing of variables from the model.

## Conclusions

Despite progress made in maternal healthcare service utilisation in Ethiopia, the country still has unsatisfactory levels of optimum utilisation of maternal healthcare services, such as having the recommended number of ANC visits. From this study, we can conclude that, in Ethiopia, as maternal healthcare service utilisation increases, the probability of infant deaths decreases. If women use optimum maternal healthcare services during pregnancy and delivery, the IMR may reduce in Ethiopia. Essential maternal healthcare service intervention packages in Ethiopia should involve a variety of care that spans pregnancy, childbirth and post-delivery, to improve survival rates of children in their first year of life. Optimising maternal healthcare services in a resource-limited setting like Ethiopia is strongly recommended to reduce infant mortality. The findings from this study may provide important information for identifying priority interventions for maternal and infant survival and for developing policies and programs that can help to achieve the SDGs. It may also have important implications for maternal and child health programs in Ethiopia.

## Data Availability

Data will be available upon reasonable request from the corresponding author. The data is available on MEASURE DHS website (www.meauredhs.com).

## Abbreviations

CSA: Central Statistics Agency
EA: Enumeration Area
EDHS: Ethiopia Demographic and Health Survey
MDGs: Millennium Development Goals
PHC: Ethiopia Population and Housing Census
SDGs: Sustainable Development Goals
USAID: United States Agency for International Development
WHO: World Health Organization
